# Supplementary material for: Variant Characterization of a Representative Large Pedigree Suggests “Variant Risk Clusters” Convey Varying Predisposition of Risk to Lynch Syndrome
Source: Cancers (Basel). 2023 Aug 12;15(16):4074. doi: 10.3390/cancers15164074 (PMC10452890; doi:10.3390/cancers15164074)
Supplement: Supplementary file 1 [file cancers-15-04074-s001.zip › cancers-2480987-supplementary.pdf]

cancers

MDPI

Variant Characterization of a Representative Large Pedigree Suggests "Variant Risk Clusters" Convey Varying Predisposition of Risk to Lynch Syndrome

Supplementary Materials

S1. Data Processing and Whole-Genome Mapping

Raw sequence data in FASTQ format was aligned to the Human Reference Genome (NCBI Build 37) using the Burrows–Wheeler Aligner (BWA) v0.7.17 [1] The alignment files, initially in SAM format, were subsequently converted to BAM format with samtools v1.9 [2]. PCR and optical duplicated reads were identified using the MarkDuplicates subroutine in Picard v2.18.9 (http://broadinstitute.github.io/picard/). Further refinement of the raw alignements was performed using the Genome Analysis Toolkit (GATK) v.3.8.1 [3]. This process involved realignment of reads (GATK's RealignerTargetCreator and IndelRealigner) and recalibration of base quality scores (GATK's BaseRecalibrator and PrintReads). An average alignment rate of 99.65% per sample was achieved, providing an approximation of 30-fold average genomic coverage.

S2. Variant Calling and Annotation

S2.1. SNPs and Indels

Single nucleotide polymorphisms (SNPs) and insertions and deletions (Indels) were called using GATK HaplotyperCaller, generating individual GVCF files for each respective sample. Subsequently, these GVCF files underwent joint genotyping by GATK GenotypeGVCFs, producing a single VCF file including all samples. A quality control procedure for this multi-sample VCF file was conducted via variant quality score recalibration (VQSR), utilizing GATK's VariantRecalibrator and ApplyRecalibration; a tranche sensitivity cutoff of 99.5% for SNPs and 99% for Indels were applied for downstream analysis. To evaluate their functional impact, variants were annotated using ANNOVAR [4] and the databases it integrates, including 1000 Genomes [5], dbSNP [6], and ExAC [7].

S2.2. Structural Variants

The smoove pipeline was employed to detect structural variants (SVs), including copy number variations (CNVs), by utilizing Lumpy [8]for calling and Svtyper [9] for genotyping SVs. Deletions and duplications were annotated with depth information using Duphold [10]. Retention of SVs was based on the criteria of a duphold flank fold-change (DHFFC) below 0.7 for deletions and a duphold bin fold-change (DHBFC) above 1.3 for duplications [11]. All deletions and duplications that met these criteria, as well as all inversions and breakends detected by smoove, were then annotated using AnnotSV [12].

S2.3. Segregation in Pedigrees

Subjects were segregated into three groups based on familial colorectal cancer (CRC) status:

Group 1: High risk to LS, (HRLS, Fig 1, red ovals) included CRC-affected subjects with one CRC-affected parent of the pedigree; Group 2: Intermediate Risk to LS (IRLS, Fig 1, green ovals) includes CRC-free subjects that have at least one affected parent, and Group 3: Low Risk to LS (LRLS, Fig 1, blue ovals) are those who with no affected in the subject's immediate triplet (subject and both parents).

S3. Variant Filtering

S3.1. SNPs and Indels

A filtration process was applied to the variants to isolate those with the highest potential for functional impact. Initially, variants with a Minor Allele Frequency (MAF) >= 1% were eliminated, as per the 1000 Genomes [5] and ExAC non-TCGA database [7]. Subsequently, the CADD database was utilized to rank the variants, with those having a PHRED scaled score of > 10 and falling within the top 10% of probable functional variants deemed as deleterious [13]. The deleterious nature of the missense coding variants was then assessed using MutationTaster [14], PolyPhen V2 [15], Provean [16], and SIFT [17], with the data sourced from dbNSFP [18]. Variants predicted as deleterious by a minimum of three of these tools underwent further analysis. The deleterious nature of non-coding variants was predicted using HaploReg V4 [19] and Regulome DB [20], primarily based on ENCODE data [21].

S3.2. Structural Variants

A filtration process was similarly applied to the SVs to isolate those with the highest potential for functional impact. Variants with a Minor Allele Frequency (MAF) >= 1% were initially excluded, in accordance with the 1000 Genomes and Genome Aggregation Database (gnomAD) [22] The AnnotSV ranking, guided by the American College of Medical Genetics and Genomics guidelines [23], was then employed to classify the variants. Variants achieving a rank of 4 (likely pathogenic) or 5 (pathogenic) were subjected to further analysis.

4. Filtered Variants According to Cancer Relationship

S4.1. SNPs and Indels

SNPs and Indels with functional impact, as identified by the described analytical workflow, underwent analysis via SNPnexus [24] to ascertain phenotype and disease associations, as well as biological clinical interpretations. The evaluation of variants took into account their association with cancer to predict their potential role in various cancers, including CRC. The Cancer Genome Interpreter database was utilized to assess these variants for oncogenic classification and tumor driver status [25]. Subsequently, the Genetic Association of Complex Diseases and Disorders database was employed to predict the association of these noted variants with cancer, based on the variant's annotation with the disease class of cancer [26].

S4.2. Structural Variants

Pathogenic or likely pathogenic Structural Variants (SVs), per the analytical workflow, were examined based on their overlapping gene. The genes identified were cross-referenced with the "Cancer Genes" list from the Precision Oncology Knowledge Base (OncoKB) [27] to determine their association with cancer. The genes that matched were further evaluated using OncoKB and My Cancer Genome [28] for their association with various cancers, including CRC.

References

1. Li, H.; Durbin, R. Fast and Accurate Long-Read Alignment with Burrows–Wheeler Transform. *Bioinformatics* **2010**, *26*, 589–595, doi:10.1093/bioinformatics/btp698.
2. Li, H.; Handsaker, B.; Wysoker, A.; Fennell, T.; Ruan, J.; Homer, N.; Marth, G.; Abecasis, G.; Durbin, R.; 1000 Genome Project Data Processing Subgroup The Sequence Alignment/Map Format and SAMtools. *Bioinformatics* **2009**, *25*, 2078–2079, doi:10.1093/bioinformatics/btp352.
3. McKenna, A.; Hanna, M.; Banks, E.; Sivachenko, A.; Cibulskis, K.; Kernytsky, A.; Garimella, K.; Altshuler, D.; Gabriel, S.; Daly, M.; et al. The Genome Analysis Toolkit: A MapReduce Framework for Analyzing next-Generation DNA Sequencing Data. *Genome Res.* **2010**, *20*, 1297–1303, doi:10.1101/gr.107524.110.

4. Wang, K.; Li, M.; Hakonarson, H. ANNOVAR: Functional Annotation of Genetic Variants from High-Throughput Sequencing Data. *Nucleic Acids Research* **2010**, *38*, e164, doi:10.1093/nar/gkq603.
5. A Global Reference for Human Genetic Variation. *Nature* **2015**, *526*, 68–74, doi:10.1038/nature15393.
6. Smigielski, E.M.; Sirotkin, K.; Ward, M.; Sherry, S.T. DbSNP: A Database of Single Nucleotide Polymorphisms. *Nucleic Acids Research* **2000**, *28*, 352–355, doi:10.1093/nar/28.1.352.
7. Lek, M.; Karczewski, K.J.; Minikel, E.V.; Samocha, K.E.; Banks, E.; Fennell, T.; O'Donnell-Luria, A.H.; Ware, J.S.; Hill, A.J.; Cummings, B.B.; et al. Analysis of Protein-Coding Genetic Variation in 60,706 Humans. *Nature* **2016**, *536*, 285–291, doi:10.1038/nature19057.
8. Linderman, M.D.; Paudyal, C.; Shakeel, M.; Kelley, W.; Bashir, A.; Gelb, B.D. NPSV: A Simulation-Driven Approach to Genotyping Structural Variants in Whole-Genome Sequencing Data. *GigaScience* **2021**, *10*, giab046, doi:10.1093/gigascience/giab046.
9. Layer, R.M.; Chiang, C.; Quinlan, A.R.; Hall, I.M. LUMPY: A Probabilistic Framework for Structural Variant Discovery. *Genome Biology* **2014**, *15*, R84, doi:10.1186/gb-2014-15-6-r84.
10. Chiang, C.; Layer, R.M.; Faust, G.G.; Lindberg, M.R.; Rose, D.B.; Garrison, E.P.; Marth, G.T.; Quinlan, A.R.; Hall, I.M. SpeedSeq: Ultra-Fast Personal Genome Analysis and Interpretation. *Nat Methods* **2015**, *12*, 966–968, doi:10.1038/nmeth.3505.
11. Pedersen, B.S.; Quinlan, A.R. Duphold: Scalable, Depth-Based Annotation and Curation of High-Confidence Structural Variant Calls. *GigaScience* **2019**, *8*, giz040, doi:10.1093/gigascience/giz040.
12. Geoffroy, V.; Herenger, Y.; Kress, A.; Stoetzel, C.; Piton, A.; Dollfus, H.; Muller, J. AnnotSV: An Integrated Tool for Structural Variations Annotation. *Bioinformatics* **2018**, *34*, 3572–3574, doi:10.1093/bioinformatics/bty304.
13. Kircher, M.; Witten, D.M.; Jain, P.; O'Roak, B.J.; Cooper, G.M.; Shendure, J. A General Framework for Estimating the Relative Pathogenicity of Human Genetic Variants. *Nat Genet* **2014**, *46*, 310–315, doi:10.1038/ng.2892.
14. Schwarz, J.M.; Rödelsperger, C.; Schuelke, M.; Seelow, D. MutationTaster Evaluates Disease-Causing Potential of Sequence Alterations. *Nat Methods* **2010**, *7*, 575–576, doi:10.1038/nmeth0810-575.
15. Adzhubei, I.A.; Schmidt, S.; Peshkin, L.; Ramensky, V.E.; Gerasimova, A.; Bork, P.; Kondrashov, A.S.; Sunyaev, S.R. A Method and Server for Predicting Damaging Missense Mutations. *Nat Methods* **2010**, *7*, 248–249, doi:10.1038/nmeth0410-248.
16. Choi, Y.; Chan, A.P. PROVEAN Web Server: A Tool to Predict the Functional Effect of Amino Acid Substitutions and Indels. *Bioinformatics* **2015**, *31*, 2745–2747, doi:10.1093/bioinformatics/btv195.
17. Ng, P.C.; Henikoff, S. SIFT: Predicting Amino Acid Changes That Affect Protein Function. *Nucleic Acids Research* **2003**, *31*, 3812–3814, doi:10.1093/nar/gkg509.
18. DbNSFP v3.0: A One-Stop Database of Functional Predictions and Annotations for Human Nonsynonymous and Splice-Site SNVs - Liu - 2016 - Human Mutation - Wiley Online Library Available online: https://onlinelibrary.wiley.com/doi/full/10.1002/humu.22932 (accessed on 17 April 2023).
19. Ward, L.D.; Kellis, M. HaploReg v4: Systematic Mining of Putative Causal Variants, Cell Types, Regulators and Target Genes for Human Complex Traits and Disease. *Nucleic Acids Research* **2016**, *44*, D877–D881, doi:10.1093/nar/gkv1340.
20. Boyle, A.P.; Hong, E.L.; Hariharan, M.; Cheng, Y.; Schaub, M.A.; Kasowski, M.; Karczewski, K.J.; Park, J.; Hitz, B.C.; Weng, S.; et al. Annotation of Functional Variation in Personal Genomes Using RegulomeDB. *Genome Res.* **2012**, *22*, 1790–1797, doi:10.1101/gr.137323.112.
21. Birney, E.; Stamatoyannopoulos, J.A.; Dutta, A.; Guigó, R.; Gingeras, T.R.; Margulies, E.H.; Weng, Z.; Snyder, M.; Dermitzakis, E.T.; Stamatoyannopoulos, J.A.; et al. Identification and Analysis of Functional Elements in 1% of the Human Genome by the ENCODE Pilot Project. *Nature* **2007**, *447*, 799–816, doi:10.1038/nature05874.
22. Collins, R.L.; Brand, H.; Karczewski, K.J.; Zhao, X.; Alföldi, J.; Francioli, L.C.; Khera, A.V.; Lowther, C.; Gauthier, L.D.; Wang, H.; et al. A Structural Variation Reference for Medical and Population Genetics. *Nature* **2020**, *581*, 444–451, doi:10.1038/s41586-020-2287-8.
23. Richards, S.; Aziz, N.; Bale, S.; Bick, D.; Das, S.; Gastier-Foster, J.; Grody, W.W.; Hegde, M.; Lyon, E.; Spector, E.; et al. Standards and Guidelines for the Interpretation of Sequence Variants: A Joint Consensus Recommendation of the American College of Medical Genetics and Genomics and the Association for Molecular Pathology. *Genet Med* **2015**, *17*, 405–423, doi:10.1038/gim.2015.30.
24. Oscanoa, J.; Sivapalan, L.; Gadaleta, E.; Dayem Ullah, A.Z.; Lemoine, N.R.; Chelala, C. SNPnexus: A Web Server for Functional Annotation of Human Genome Sequence Variation (2020 Update). *Nucleic Acids Research* **2020**, *48*, W185–W192, doi:10.1093/nar/gkaa420.
25. Becker, K.G.; Barnes, K.C.; Bright, T.J.; Wang, S.A. The Genetic Association Database. *Nat Genet* **2004**, *36*, 431–432, doi:10.1038/ng0504-431.
26. Tamborero, D.; Rubio-Perez, C.; Deu-Pons, J.; Schroeder, M.P.; Vivancos, A.; Rovira, A.; Tusquets, I.; Albanell, J.; Rodon, J.; Tabernero, J.; et al. Cancer Genome Interpreter Annotates the Biological and Clinical Relevance of Tumor Alterations. *Genome Med* **2018**, *10*, 25, doi:10.1186/s13073-018-0531-8.
27. Chakravarty, D.; Gao, J.; Phillips, S.; Kundra, R.; Zhang, H.; Wang, J.; Rudolph, J.E.; Yaeger, R.; Soumerai, T.; Nissan, M.H.; et al. OncoKB: A Precision Oncology Knowledge Base. *JCO Precision Oncology* **2017**, 1–16, doi:10.1200/PO.17.00011.
28. Home—My Cancer Genome Available online: https://www.mycancergenome.org/ (accessed on 12 June 2023).
